# CCK1-Receptor Stimulation Protects Against Gut Mediator-Induced Lung Damage During Endotoxemia

**DOI:** 10.1159/000356644

**Published:** 2013-12-20

**Authors:** Friederike Eisner, Elizabeth M. Martin, Markus A. Küper, Helen E. Raybould, Jörg Glatzle

**Affiliations:** a Department of General, Visceral and Transplant Surgery, University Hospital of Tuebingen, Tuebingen, Germany; b Department of Anatomy, Physiology and Cell Biology, School of Veterinary Medicine, University of California Davis, Davis, CA, USA

**Keywords:** Sepsis, Endotoxemia, Innate immunity, Mucosal immunity, Cholinergic anti-inflammatory pathway, Cholecystokinin

## Abstract

**Background/Aims:**

Cholecystokinin 1-receptor (CCK1-R) activation by long chain fatty acid (LCFA) absorption stimulates vago-vagal reflex pathways in the brain stem. The present study determines whether this reflex also activates the cholinergic anti-inflammatory pathway, a pathway known to modulate cytokine release during endotoxemia.

**Methods:**

Mesenteric lymph was obtained from wild type (WT) and CCK1-R knockout (CCK1-R^−/−^) mice intraperitoneally challenged with Lipopolysaccharid (LPS) (endotoxemic lymph, EL) and intestinally infused with vehicle or LCFA-enriched solution. The lymph was analyzed for TNFα, IL-6 and IL-10 concentration and administered to healthy recipient mice via jugular infusion. Alveolar wall thickness, myeloperoxidase (MPO) and TUNEL positive cells were determined in lung tissue of recipient mice.

**Results:**

LCFA infusion in WT mice reduced TNFα concentration in EL by 49% compared to vehicle infusion, but had no effect in CCK1-R^−/−^ mice. EL significantly increased the alveolar wall thickness, the number of MPO-positive and TUNEL-positive cells compared to control lymph administration. LCFA infusion in WT, but not in CCK1R^−/−^ mice, significantly reduced these pathological effects of EL.

**Conclusion:**

During endotoxemia enteral LCFA absorption reduces TNFα release into mesenteric lymph and attenuates histomorphologic parameters of lung dysfunction. Failure to elicit this effect in CCK1R^−/−^ mice demonstrates that anti-inflammatory properties of LCFAs are mediated through CCK1-Rs.

## Introduction

In spite of all advances in antimicrobial therapy and intensive care treatment, severe sepsis and septic shock remain a major challenge on intensive care units and are a common cause of death in industrialized countries [1, 2].

Sepsis is a heterogeneous and dynamic disease characterized by the systemic manifestation of inflammation induced by a microorganism [3, 4]. The crucial step for the rapid initiation of host defense responses after microbial infection is generally considered to be the activation of pattern-recognition receptors (PRR) such as toll-like receptors (TLR) [5–7]. During sepsis, the release of high levels of pathogen-associated molecular patterns (PAMP) and/or intracellular proteins and molecules from dying cells (so called alarmins [5]) induce an overwhelming activation of mediators, free radicals and enzymes. Thereby, the normally protective inflammatory response grows to a severe life-threatening inflammatory state [8]. With loss of regulatory function, the local response can lead to organ dysfunction at sites far from the initial site of infection. This condition, referred to as severe sepsis [4], most frequently effects the respiratory system, followed by cardiovascular dysfunction, coagulation disorders, renal failure, CNS derangements, and hepatic dysfunction [1]. A progressive dysfunction in two or more organ systems is referred to as multiple organ dysfunction syndrome (MODS) [3, 9].

Signs of a systemic inflammation, similar to those observed during sepsis, can develop in the absence of an invasive infection e.g. in pancreatitis or severe trauma. This finding led to the concept of a systemic inflammatory response syndrome (SIRS) [3, 4]. Likewise the toxic effect of Lipopolysaccharide (LPS) is due to a powerful immune response triggered by the LPS - leukocyte interaction eventually resulting in shock and MODS [7, 10]. LPS, also referred to as endotoxin, is an important component of the outer membrane of Gram-negative bacteria [10]. LPS signaling is mediated by TLR4, a member of the TLR family expressed on the surface of antigen-presenting cells such as macrophages, dendritic cells and B cells [7, 11, 12]. Ligand binding to TLR4 results in synthesis and release of inflammatory cytokines [7, 13]. It has been demonstrated, that the pro-inflammatory cytokines Interleukin (IL)-1 and tumor necrosis factor (TNF) α can elicit pathological responses similar to clinical features of sepsis [14]. All together these observations support the concept of host-derived non-bacterial factors as major cause of the MODS [4, 15].

The gut associated lymphoid tissue (GALT) is the largest lymphoid organ [16]. The associated immune cells are unique in their properties which enable both tolerance to huge amounts of microbes and antigens as well as protection against harmful pathogens [16, 17]. Disruption of the intestinal integrity by infection, ischemia or trauma expeditiously results in SIRS or sepsis eventually leading to MODS [15, 18]. A pivotal role of mesenteric lymph in dispersing pro-inflammatory factors during experimental endotoxemia [19, 20], trauma and hemorrhagic shock [21–23] has been shown recently. Immune cells and pro-inflammatory mediators are released from the gut wall into mesenteric lymph and drained into the systemic circulation via the thoracic duct. Thus, the pulmonary vascular bed is the first vascular bed exposed to mediators carried by mesenteric lymph [18]. Severe sepsis, major trauma with hemorrhage and shock as well as severe pancreatitis are frequent causes of acute lung injury (ALI) and the acute respiratory distress syndrome (ARDS) [1, 24]. An incidence of about 200,000 people annually with approximately 40% fatal outcome is reported in the United States [24]. In experimental models of trauma, hemorrhagic shock and burns lung injury could be reduced or even prevented by mesenteric lymph duct division [25–28] accounting for mesenteric lymph as primary route for gut-derived inflammatory factors.

Recent studies have revealed the prominent role of autonomic control of the immune system in inflammatory disease. Information about locally released inflammatory mediators is transmitted to the central nervous system (CNS) via afferent nerves and the bloodstream. As a result, the CNS modulates immune responses through complex integration of neural, cytokine, and hormonal inputs, resulting in efferent vagal function forming the so called cholinergic anti-inflammatory pathway [29, 30]. The idea of a cholinergic anti-inflammatory pathway was first formulated by Tracey and colleagues, who determined that electrical stimulation of the cervical vagal nerve before and after intravenous LPS administration diminished both TNFα levels in serum and liver and the development of septic shock [29]. *In vitro* culture of isolated human macrophages demonstrated that acetylcholine reduced the release of pro-inflammatory cytokines TNFα, IL-6, IL-1β, IL-18 but had no effect on anti-inflammatory IL-10 levels [29]. They identified the nicotinic acetylcholine receptor alpha 7 (nα7AChR) to be present in macrophages and responsible for the anti-inflammatory effect observed [31].

There is evidence for the ability of macronutrients to modulate the systemic inflammatory response via the autonomic nervous system. The interaction of nutrition and the nervous system is known to be crucial for the regulation of gut motility and digestion as well as food intake and satiety. Investigation into the role of nutrient modulation of the cholinergic anti-inflammatory pathway has shown that ingestion of lipids attenuated TNFα serum levels in rodent models of hemorrhagic shock [32] and endotoxemia [33]. Previously, we demonstrated that ingestion of long chain fatty acids (LCFA) decreased TNFα levels in the mesenteric lymph and reduced the damaging effect of endotoxemic mesenteric lymph to the lung tissue in a rat model [19]. The observation, that vagotomy as well as a combined antagonism of cholecystokinin 1- and cholecystokinin 2-receptors (CCK1-R and CCK2-R) counteract the protective lipid effect [32, 33] provided first hints about the involvement of the vagal afferent pathway. Fatty acids with a chain length of ten or more carbon atoms trigger the release of the peptide hormone cholecystokinin (CCK) from specialized enteroendocrine cells in the proximal small intestine. Vagal afferent nerve terminals, expressing predominantly CCK1-R, are located in immediate adjacency to CCK-producing cells and are activated by CCK [34].

At present, there is lack of evidence for the critical role of CCK1-Rs in mediating the anti-inflammatory impact of enteral lipid absorption. It also remains unclear whether the release of cytokines, in addition to TNFα, into mesenteric lymph is influenced by absorption of LCFA. The present study uses a CCK1-R knockout mouse model of endotoxemia to address these questions.

## Material and Methods

### Animals

Experiments were performed using male 129S6/SvEv mice (hereafter referred to as WT, Taconic Farms, Oxnard, CA and CCK1-Receptor knockout mice (hereafter referred to as CCK1-R^−/−^ mice, 129S6/SvEv background) of 20–30g weight. CCK1-R^−/−^ mice display normal body weight and are normoglycemic [35]. Animals were maintained on commercially available laboratory chow (Purina Laboratory, diet #5001, St. Louis, MO) and were housed under controlled conditions of illumination (12:12h light/ dark cycle starting at 6 p.m.), humidity, and temperature (21°C) with free access to food and water. Before surgical procedures, animals were fasted for 18 hours but allowed water and a 5% glucose solution *ad libitum*. Institutional guidelines for the care and use of laboratory animals were followed throughout the study.

### Mesenteric lymph collection

The method of mesenteric lymph duct cannulation was previously published [36]. In brief, animals were anesthetized using a combination of Isoflurane (Piramal Healthcare, India) and i.p. Methohexital Sodium (50mg/kg BW, JHP Pharmaceuticals, USA). A laparotomy was performed through a midline incision, the superior mesenteric lymph duct was identified using a microscope, and a polyurethane tube was inserted into the lymph duct (Micro-Renathane, 0.64mm O.D. x 0.30mm I.D., Braintree Scientific, Inc., USA). The tube was fixed in place with a drop of cyanoacryl glue (Krazy Glue, Elmer’s Products Inc., USA) and externalized through an incision in the right flank. A second catheter (Silastic, 1.65mm O.D. x 0.76mm I.D., Dow Corning Corporation, USA) was placed into the duodenum through the fundus of the stomach, fixed with a polypropylene suture, and externalized through the left flank. Buprenorphine (0.05mg/ kg BW, s.c., Reckitt Benckiser Pharmaceuticals Inc., USA) was administered postoperatively. To prevent catheters from dislocation, mice were placed in modified Bollman cages after surgery. A glucose-electrolyte solution (Glucose 0.2mol/L, NaCl 145 mmol/L, and KCl 4mmol/L with or without 1% ClinOleic, a mixture of 80% olive oil and 20% soybean oil, Baxter, Germany) was infused continuously through the duodenal cannula at a rate of 0.5ml/h. Mice were allowed to recover from surgery for 12 hours while the mesenteric lymph was drained freely and mice were intestinally infused as above mentioned. Thereafter, mesenteric lymph was collected for two hours before (control lymph) and for a six hours after i.p. LPS injection (LPS, Escherichia coli serotype O111:B4, Sigma, USA, 5mg/kg body weight in 0.2ml, endotoxemic lymph) from four different experimental groups (n=8 per group):

- WT mice, intestinally infused with vehicle solution (Glucose 0.2mol/L, NaCl 145 mmol/L, and KCl 4mmol/L).
- WT mice, intestinally infused with long chain fatty acid (LCFA) solution (1% ClinOleic, Glucose 0.2mol/L, NaCl 145 mmol/L, and KCl 4mmol/L).
- CCK1-R^−/−^ mice, intestinally infused with vehicle solution (Glucose 0.2mol/L, NaCl 145 mmol/L, and KCl 4mmol/L).
- CCK1-R^−/−^ mice, intestinally infused with LCFA solution (1% ClinOleic, Glucose 0.2mol/L, NaCl 145 mmol/L, and KCl 4mmol/L).

Lymph was collected in two hour time intervals in ice-chilled tubes, centrifuged at 2000g, frozen and stored at −80°C for further experiments.

### Lymph cytokine determination

Concentration of TNFα, IL-6 and IL-10 was determined by multiplex bead-based assays (Bio-Plex, Bio-Rad, USA) according to the manufacturer’s protocol. Briefly, 50μl aliquots of diluted lymph samples and cytokine standards were incubated in a 96-well filter plate with capture antibody-coupled beads for 30min at room temperature while protected from light and with modest shaking. After three washing steps detection antibodies were added and incubated for 30min. Samples were washed and 10min incubated with Streptavidin-PE. Thereafter samples were washed and re-suspended in Assay Buffer (Bio-Rad, USA). The Bio-Plex System (Luminex xMAP-Technology, Bio-Rad, USA) was used for data acquisition and cytokine concentration were calculated using the Bio-Plex Manager software, version 4.0 (Bio-Rad, USA).

### Mesenteric lymph infusion

Mesenteric lymph samples of eight donor mice from each of the groups I–IV were pooled for the collection period before LPS administration (control lymph) and after LPS administration (endotoxemic lymph). The lymph was then infused (0.25ml/h, 90min) in separate healthy recipient WT mice through a catheter in the jugular vein (Micro-Renathane, 0.64mm O.D. x 0.30mm I.D., Braintree Scientific, Inc., USA).

Mesenteric lymph infusion was performed in 6 different experimental groups:

- WT mice control lymph (CL): Infusion of lymph obtained from WT mice intestinally infused with vehicle solution (n=4).
- WT mice Endotoxemic lymph (EL): Infusion of lymph obtained from WT mice intestinally infused with vehicle solution (n=6).
- WT mice EL LCFA: Infusion of lymph obtained from WT mice intestinally infused with LCFA solution and LPS administration (n=5).
- CCK1-R^−/−^ mice CL: Infusion of lymph obtained from CCK1-R^−/−^ mice intestinally infused with vehicle solution (n=4).
- CCK1-R^−/−^ mice EL: Infusion of lymph obtained from CCK1-R^−/−^ mice intestinally infused with vehicle solution and LPS administration (n=6).
- CCK1-R^−/−^ mice EL LCFA: Infusion of lymph obtained from CCK1-R^−/−^ mice intestinally infused with LCFA solution and LPS administration (n=6).

### Histological Analysis of Lung Tissue

The lung of the recipient mice was harvested immediately after the termination of lymph infusion and fixed in 4% PBS-paraformaldehyde. The fixed lung tissue was then embedded in paraffin. Paraffin sections (1μm) were deparaffinized stepwise and incubated with Hematoxylin and Eosin (H&E), Myeloperoxidase (MPO), or the In-Situ-Cell-Death-Detection Kit (POD, Roche, Penzberg, Germany).

To determine the thickness of the alveolar walls the histological sections were stained with H&E. The diameter of 30 alveolar walls was determined in one optical section (W: 650pp, F: 515pp) using the Quantimet System (Leica, magnification x 400). Ten optical sections were analyzed for each animal.

For MPO immunohistochemistry and the TUNEL reaction histological sections were pre-incubated with 30% hydrogen peroxide in methanol for 15 minutes in order to block the endogenous peroxidase. By incubation in 20% swine serum for 20 minutes nonspecific background staining was blocked. The tissue was then incubated overnight with a rabbit anti-MPO antibody (1:50, Dianova, Germany) at room temperature. Thereafter specimens were washed in phosphate-buffered saline and for 60 minutes incubated with a biotinylated, swine anti-rabbit antibody (1:600, DAKO, Germany) at room temperature. The avidin-biotin complex (ABC) method was used to demonstrate MPO immunoreactivity with 3,3′ diaminobenzidine 0.05%/hydrogen peroxide 0.033% (DAB) serving as chromagen. For each animal MPO positive cells were determined in 30 optical sections.

The In-Situ-Cell-Death-Detection Kit (Roche, Germany) was used for detection of apoptotic cells following the manufacturer’s instruction. In short, after blocking of the endogenous peroxidase histological sections were five minutes pretreated with microwave irradiation (700 Watt) in 0.01M citrate buffer (pH 6.0). Background was diminished by preincubating with 3% bovine serum albumin (BSA) in 0.1M Tris-HCl. Afterwards the specimens were incubated for one hour at 37°C with the TUNEL labeling mix. POD was used for signal conversion and TUNEL positive cells were demonstrated by the DAB color reaction. For each animal TUNEL positive cells were determined in 30 optical sections.

For the statistical calculation the average value of the thickness of the alveolar walls, number of MPO-positive cells and TUNEL-positive cells of all optical sections of one specimen were used as a single value.

### Statistical analyses

Data are presented as mean ± standard error of the mean (SEM). Differences between the groups were determined by unpaired Student’s t-test using the software package of GraphPad Prism 4.0 (San Diego, CA). A probability of p<0.05 was taken as significant.

## Results

### Mesenteric lymph flow

**Fig. 1:**
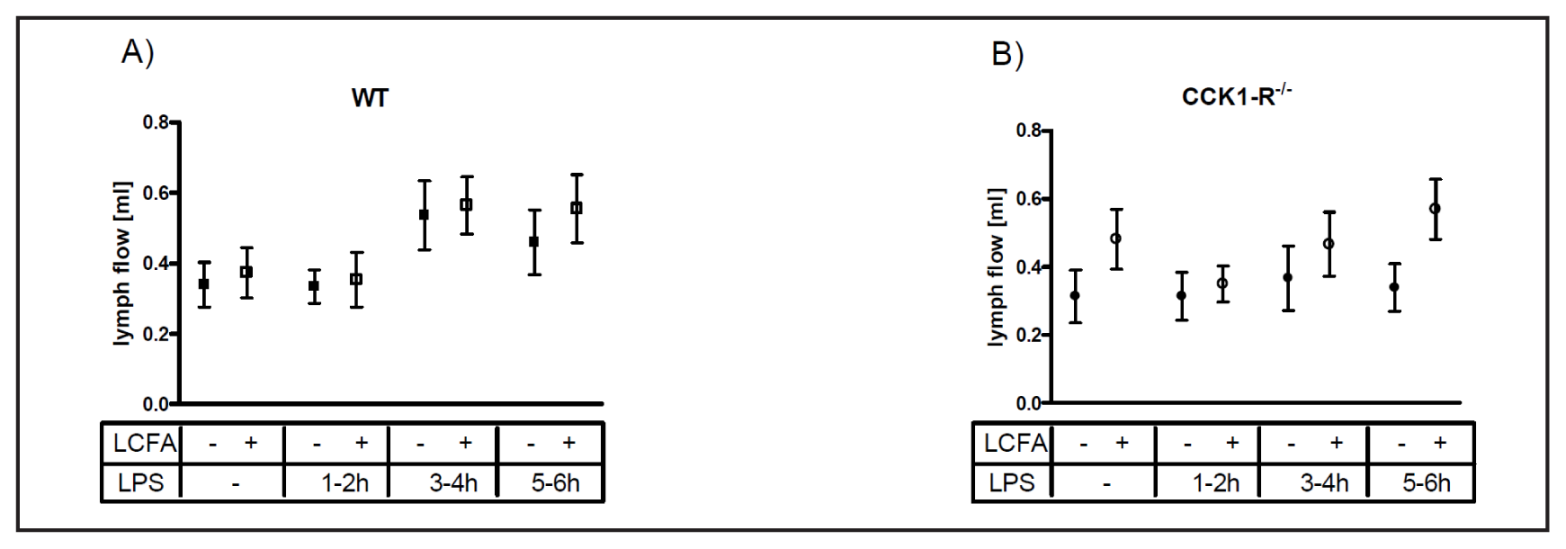
Mesenteric lymph flow (ml) was determined in two hour (h) time intervals before and after i.p. LPS administration (5mg/kg body weight) in A) wild type (WT) mice and B) CCK1-receptor knockout (CCK1-R^−/−^) mice. Mesenteric lymph flow was affected by neither LPS administration nor intestinal long chain fatty acid absorption.

The amount of mesenteric lymph collected in the two hour time intervals was between 0.3ml and 0.6ml. There was no significant effect of treatment with LPS or intestinal LCFA on lymph flow (Fig. 1).

### LPS challenge provoked a cytokine release into mesenteric lymph which differed between WT and CCK1-R^−/−^ mice

**Fig. 2:**
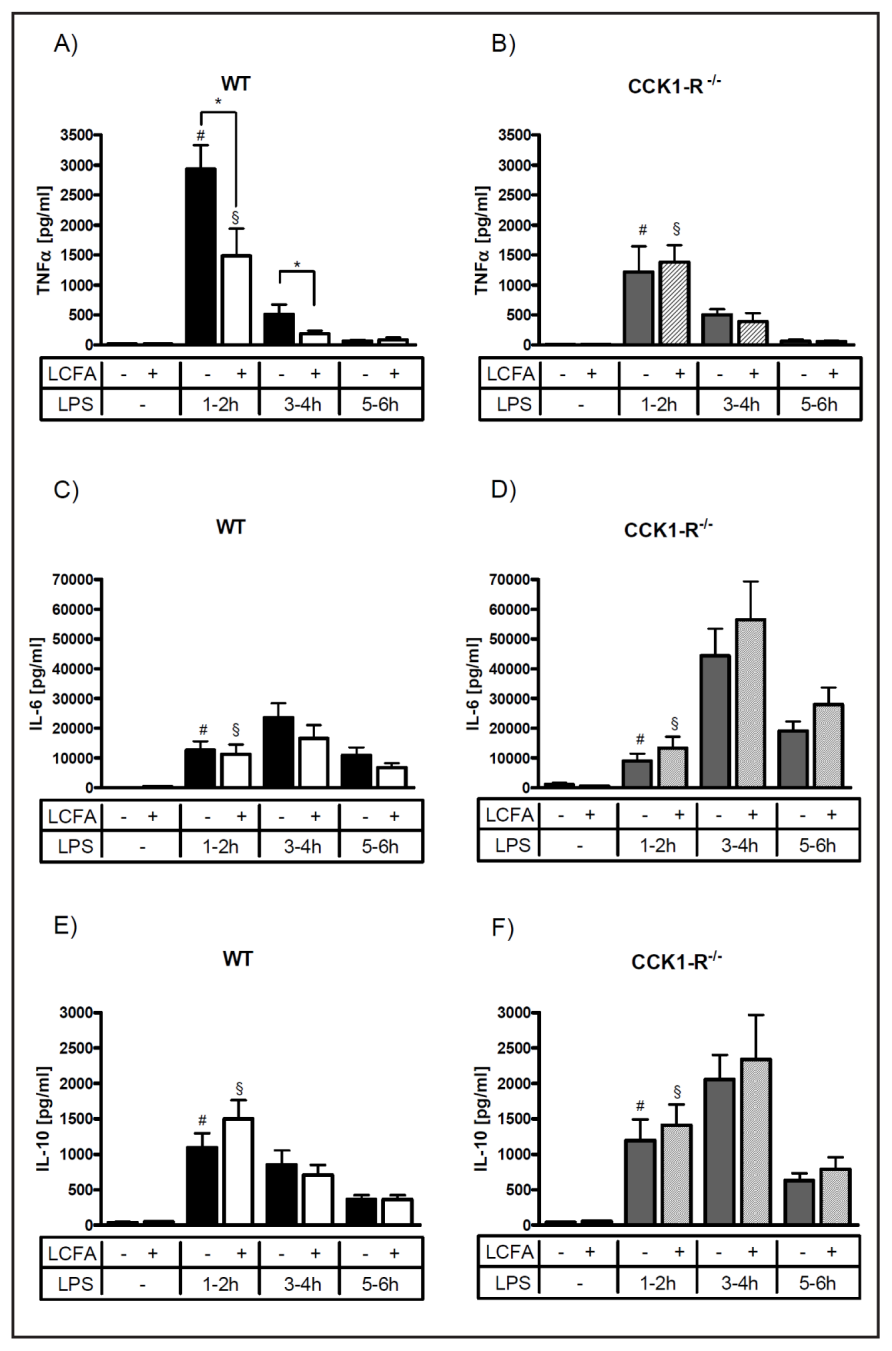
Cytokine concentration in mesenteric lymph was determined before and after LPS challenge in both genotypes intestinally infused with either vehicle or long chain fatty acid solution. LPS challenge provoked a cytokine release into mesenteric lymph which differed between WT and CCK1-R^−/−^ mice. Intestinal long chain fatty acid (LCFA) absorption decreased TNFα release into mesenteric lymph in WT mice but not in CCK1-R^−/−^ mice. IL-6 and IL-10 concentration were not affected by LCFA absorption. Mesenteric lymph concentration of A) TNFα in wild type (WT) mice; B) TNFα in CCK1-R knockout (CCK1-R^−/−^) mice; C) IL-6 in WT mice; D) IL-6 in CCK1-R^−/−^ mice; E) IL-10 in WT mice and F) IL-10 in CCK1-R^−/−^ mice before and up to six hours (h) after i.p. LPS administration (5mg/kg body weight) was determined via multi-bead ELISA. * p<0.05 vehicle infusion vs. LCFA infusion, ^#^ p<0.05 control lymph vs. endotoxemic lymph collected during vehicle infusion, ^§^ p<0.05 control lymph vs. endotoxemic lymph collected during LCFA infusion.

Before LPS administration, the concentration of pro-inflammatory TNFα and anti-inflammatory IL-10 in mesenteric lymph was similar in WT and CCK1-R^−/−^ mice (TNFα: 19 ± 5 pg/ml vs. 11 ± 4 pg/ml; IL-10: 37 ± 12 pg/ml vs. 38 ± 11 pg/ml, WT and CCK1-R^−/−^ mice respectively; not significant). In contrast, the concentration of pro-inflammatory IL-6 was lower in WT mice (96 ± 37 pg/ml) compared to CCK1-R^−/−^ mice (1166 ± 473 pg/ml) (p<0.05) (Fig. 2).

In both genotypes, LPS treatment increased the release of TNFα, IL-6 and IL-10 into the mesenteric lymph. However, time to peak concentrations, as well as maximal concentration reached differed between the cytokines.

Maximal TNFα levels were reached in the first two hours after LPS challenge. TNFα concentrations increased 154 fold in WT mice (p<0.05) and 113 fold in CCK1-R^−/−^ mice from baseline (p<0.05) in the first two hours after LPS application. TNFα rapidly dropped down three to four hours after LPS administration in both genotypes.

IL-6 concentration in the mesenteric lymph peaked three to four hours and decreased moderately to a still marked elevation five to six hours post LPS challenge. WT mice exhibited a 246-fold increase of IL-6 concentration (p<0.05), whereas in CCKR1^−/−^ animals, IL-6 only rose to 38-fold from baseline (p<0.05). Although WT mice exhibited a greater IL-6 excursion, total IL-6 concentration was higher in CCK1-R^−/−^ mice (CCK1-R^−/−^: 44330 ± 9165 pg/ml vs. WT: 23560 ± 4865 pg/ml, 3–4 hours after LPS injection).

IL-10 concentration in the mesenteric lymph increased 30-fold in WT mice and 32-fold in CCK1-R^−/−^ mice during the first two hours after LPS challenge (p<0.05). Three to four hours after LPS application IL-10 concentration was increased by 54-fold in CCK1-R^−/−^ mice reaching peak concentrations, whereas in WT mice the IL-10 concentration was already decreasing. (Fig. 2)

### Enteral long chain fatty acid absorption in WT mice reduced the amount of TNFα released into the mesenteric lymph but had no effect in CCK1R−/− mice

The baseline concentration of pro-inflammatory TNFα and IL-6 as well as anti-inflammatory IL-10 in the mesenteric lymph was not affected by enteral LCFA absorption in both genotypes.

Absorption of LCFA reduced the TNFα release into mesenteric lymph by 49% in the first two hours after LPS challenge (p<0.05) and by 63% during the three and four hour time period in WT mice (p<0.05). In contrast LCFA absorption had no influence on the TNFα concentration in the mesenteric lymph harvested from mice lacking CCK1-R.

Enteral LCFA had no effect on lymphatic IL-6 or IL-10 concentrations after LPS challenge in either WT mice or CCK1R^−/−^ mice (Fig. 2).

### Jugular infusion of endotoxemic lymph provoked an inflammatory reaction in the lung of healthy recipient mice which was attenuated by CCK1-R activation

**Fig. 3:**
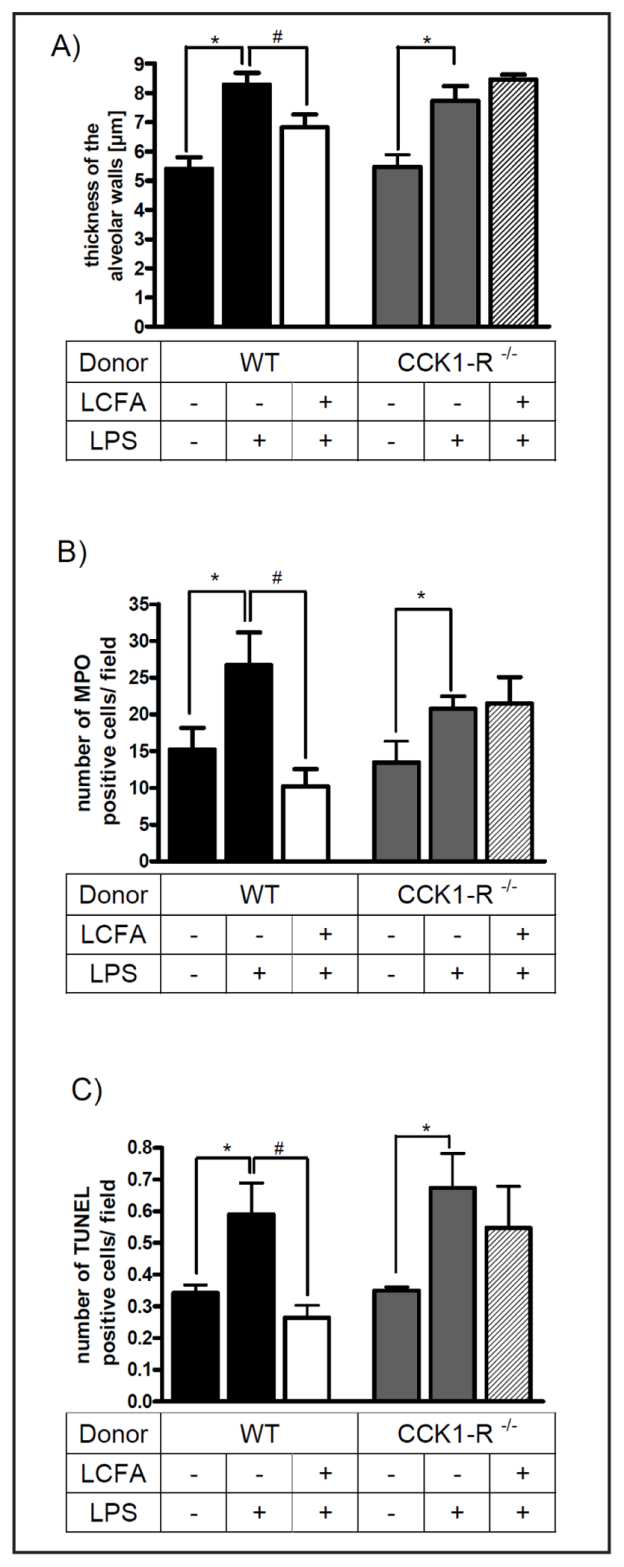
Histomorphologic parameters of lung dysfunction were determined after jugular infusion of mesenteric lymph in lung tissue of recipient wild type mice. Jugular infusion of endotoxemic lymph provoked an inflammatory reaction in the lung of recipient mice. Long chain fatty acid (LCFA) absorption during endotoxemia in WT donor mice attenuated the lung damage. In contrast LCFA absorption during endotoxemia in donor mice lacking CCK1-R did not prevent inflammatory lung damage. A) thickness of the alveolar walls, B) number of MPO positive cells and C) number of TUNEL positive cells in lung tissue of wild type (WT) mice after jugular infusion of mesenteric lymph. Lymph was harvested from (I) WT mice intestinally infused with vehicle solution, (II) WT mice intestinally infused with vehicle solution during endotoxemia, (III) WT mice intestinally infused with LCFA solution during endotoxemia, (IV) CCK1-R knockout (CCK1-R^−/−^) mice intestinally infused with vehicle solution, (V) CCK1-R^−/−^ mice intestinally infused with vehicle solution during endotoxemia, and (VI) CCK1-R^−/−^ mice intestinally infused with LCFA solution during endotoxemia. * p<0.05 control lymph vs. endotoxemic lymph, ^#^ p<0.05 vehicle infusion vs. LCFA infusion.

**Fig. 4:**
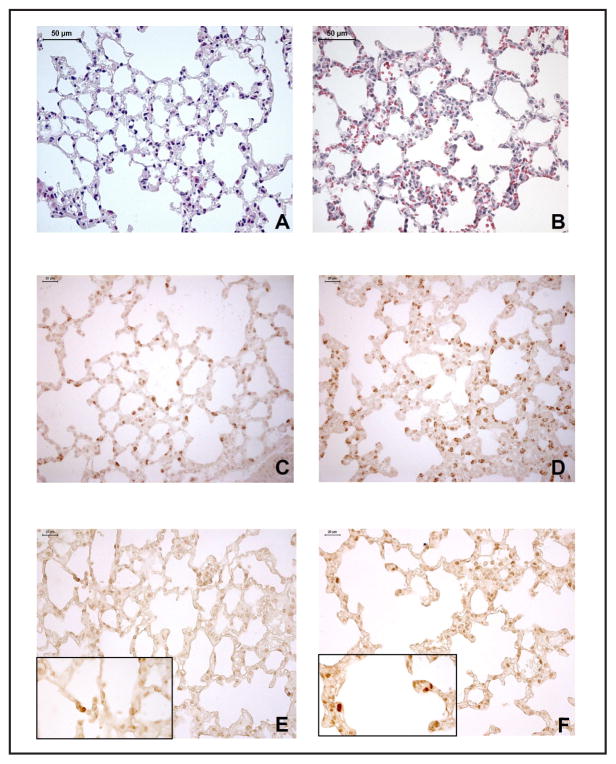
Representative sections of paraffin embedded lung tissue of recipient WT mice after jugular infusion of mesenteric lymph harvested during endotoxemia and enteral LCFA absorption. (A) and (B) H&E section for measurement of alveolar wall thickness, (A) lymph donor: WT mice; (B) lymph donor: CCK1-R^−/−^ mice. (C) and (D) Myeloperoxidase (MPO) staining of neutrophils, (C) lymph donor: WT mice; (D) lymph donor: CCK1-R^−/−^ mice. (E) and (F) detection of apoptosis by TUNEL assay, (E) lymph donor: WT mice; (F) lymph donor: CCK1-R^−/−^ mice. Original magnification, A–F x 400, details in E and F x 1,000.

Jugular infusion of EL compared to jugular infusion of CL derived from WT mice (Table 1) increased the thickness of the alveolar walls in the lung of healthy WT recipient mice by 53% (p< 0.05), the number of MPO positive cells by 75% (p< 0.05) and the number of TUNEL-positive cells by 73% (p< 0.05). EL collected from CCK1R^−/−^ mice likewise caused an augmentation in the thickness of the alveolar walls (41%, p<0.05), MPO positive cells (54% p<0.05) and TUNEL-positive cells (92% p<0.05) in healthy recipient WT mice compared to CL collected from CCK1R^−/−^ mice (Fig. 3, Fig. 4).

Jugular infusion of EL harvested from WT mice during enteral LCFA absorption significantly attenuated the increase of the alveolar wall thickness (p<0.05), the number of MPO positive cells (p<0.05) and the number of TUNEL positive cells (p<0.05) compared to jugular infusion of endotoxemic lymph harvested from WT mice during enteral infusion of vehicle solution (Fig. 3, Fig. 4). In contrast, jugular infusion of EL harvested from CCK1-R^−/−^ mice during enteral LCFA absorption had a similar effect on the thickness of the alveolar walls, the number of MPO positive cells and the number of TUNEL positive cells as jugular infusion of EL harvested from CCK1-R^−/−^ mice with enteral infusion of vehicle solution (Fig. 3, Fig. 4).

## Discussion

The GALT is considered to contain the majority of immune cells in the human body [16]. Nonbacterial, gut-derived factors are transported via mesenteric lymph into the systemic circulation [18]. A number of studies indicate the pivotal role of intestinal lymphatics in the development of ARDS and MODS [20, 27, 37]. Interruption of mesenteric lymph flow abrogates lung injury and inflammation in hemorrhagic shock [26, 27, 38] and endotoxemia [20]. Recently we have shown that enteral absorption of LCFA diminished the harmful effects of mesenteric lymph in rats challenged with LPS [19, 39]. The present study confirmed this finding in a mouse model and revealed insights into the involved pathway.

We used intraperitoneal LPS injection to induce endotoxemia, a model that permits high standardization and accurate dosing. To recognize lung injury we focused on parameters frequently used in animal models, resembling histological alterations of human ARDS [18, 40]: alveolar wall thickness as a measure for the distance of oxygen perfusion, quantification of MPO positive cells as measure of inflammation and the number of apoptotic cells as a marker for permanent tissue damage. All parameters of lung injury increased in recipient mice after jugular infusion of mesenteric lymph harvested during endotoxemia. Enteral LCFA absorption during lymph drainage in WT mice attenuated this harmful effect of EL. In contrast, enteral LCFA absorption in mice lacking CCK1-R did not prevent lung damage in recipient mice. Hence, CCK1-R activation is pivotal for the beneficial effect of LCFA absorption during endotoxemia.

CCK1-Rs are expressed by vagal afferents in the gut wall. Their activation results in signaling to neurons in the nucleus of the solitary tract (NTS) [34, 41–43]. NTS neurons project to the dorsal motor nucleus of the vagus (DMV) and other central areas. Parasympathetic DMV neurons activate nicotinic acetylcholine receptors expressed by postganglionic neurons in the gastrointestinal tract, thereby modulating motility, tone and secretion [44]. But nicotinic acetylcholine receptors are also detected on immune cells [31, 45]. Activation of the alpha 7 nicotinic acetylcholine receptors (nα7AChR) subtype diminishes NF-κB translocation into the nucleus and subsequent synthesis of pro-inflammatory cytokines like TNFα [46].

We assumed that TNFα, IL-6 and IL-10 form an important fraction of gut derived nonbacterial factors, transported via mesenteric lymph and contributing to lung injury during endotoxemia. TNFα provokes SIRS and is regarded as a key mediator of septic shock and the related MODS [47]. IL-6 induces the acute phase response and lymphocyte differentiation [48, 49] but may also possess anti-inflammatory properties [49–51]. IL-10 is considered as mainly anti-inflammatory acting and its depression is associated with uncontrolled systemic inflammatory responses [52]. Intraperitoneal injected LPS strongly increased TNFα, IL-6 and IL-10 concentration in mesenteric lymph. In WT mice enteral LCFA absorption attenuated TNFα secretion into mesenteric lymph by almost 50%, confirming previous findings in rats [19, 53]. In contrast, intestinal infusion of LCFA had no effect on TNFα release in CCK1-R^−/−^ mice, demonstrating the crucial role of CCK1-R activation. It has been suspected before that enteral lipid absorption modulates inflammation by cholecystokinin receptor (CCK-R) stimulation. The combined chemical blockage of both CCK-R subtypes (CCK1-R, CCK2-R) in rodent models of endotoxemia and hemorrhagic shock abrogates the lipid induced inhibition of TNFα release into plasma [32, 33]. Afferent fibers of the vagal nerve express CCK1-R in a three-fold higher concentration than CCK2-R [54]. Therefore we assumed that CCK1-Rs are the pivotal receptor subtype mediating the anti-inflammatory lipid effect. The abolished effect of LCFA absorption in CCK1-R^−/−^ mice, observed here, clearly strengthens this hypothesis. To our knowledge, the present study is the first one revealing that CCK1-R stimulation is the crucial factor for attenuation of inflammatory responses by enteral LCFA absorption.

The observation that enteral LCFA absorption did not alter IL-6 and IL-10 concentration in mesenteric lymph is in accordance with data presented by Rosas-Ballina et al.: TNFα, but not IL-6 and IL-10 levels in human plasma are effected by selective α7 agonism after LPS challenge [55]. Both cytokines are produced by a broad array of cell types [48, 49, 52], possibly explaining the absence of a significant inhibition by nα7AChR stimulation.

In CCK1-R^−/−^ mice LPS induced a pattern of cytokine release into mesenteric lymph which differed from the response in WT mice. TNFα increase in the first two hours after LPS application was lower than in WT mice. In contrast IL-6 and IL-10 levels were higher in mesenteric lymph of CCK1-R^−/−^ mice three to four hours after LPS application, confirming a strong innate immune response. Prior to LPS challenge IL-6 synthesis by CCK1-R^−/−^ mice exceeded the level of WT mice. Both, IL-6 and IL-10 have been shown to suppress TNFα release in experimental conditions [50, 51, 56]. Higher IL-6 levels might explain lower TNFα levels in CCK1-R^−/−^ mice. However, the reason for the pronounced IL-6 production in mice lacking CCK1-R remains unexplained. In summary, these data indicate immunological differences between CCK1-R^−/−^ mice and WT mice but further attempts are needed for clarification.

Taken together, the present study demonstrates the specific role of CCK1-R stimulation in modulating the release of TNFα into mesenteric lymph. Probably further, currently not identified mediators in the mesenteric lymph are influenced by CCK1-R stimulation and contribute to the inhibition of endotoxemia induced lung damage. Although models of endotoxemia only partially reproduce features of human sepsis our data clearly support the concept of immunomodulation by specific nutrients and the benefit of enteral feeding.

## Conclusion

Intestinal absorption of LCFA reduces the release of pro-inflammatory mediators into mesenteric lymph during endotoxemia and therefore attenuates the harmful effect of endotoxemic lymph on the lung. The anti-inflammatory effect of LCFA depends on the presence of CCK1-R. CCK1-R stimulation by a diet rich in LCFA, could provide an inexpensive and simplistic tool to prevent overwhelming inflammatory responses in critically ill patients.

## Figures and Tables

**Table 1:** Concentration of cytokines in pooled mesenteric lymph samples before and 1–6 hours after LPS-challenge

Donor	WT			CCK1-R−/−		
LCFA	−	−	+	−	−	+
LPS	−	+	+	−	+	+
TNFα [pg/ml]	21,43	1134,58	581,93	10,74	642,87	641,38
IL-6 [pg/ml]	95,98	14548,52	10931,56	1165,61	22124,73	31343,86
IL-10 [pg/ml]	35,44	771,90	909,83	37,97	1322,26	1468,26
